# Seroprevalence of Anti-S1-RBD Antibodies in Pre-pandemic and Pandemic Subjects From Hail Region, KSA

**DOI:** 10.3389/fpubh.2022.874741

**Published:** 2022-06-09

**Authors:** Subuhi Sherwani, Mohd Wajid Ali Khan, Arshi Mallik, Mahvish Khan, Mohd Saleem, Mohamed Raafat, Ayed A. Shati, Noor Alam

**Affiliations:** ^1^Department of Biology, College of Sciences, University of Ha'il, Ha'il, Saudi Arabia; ^2^Department of Chemistry, College of Sciences, University of Ha'il, Ha'il, Saudi Arabia; ^3^Department of Clinical Biochemistry, College of Medicine, King Khalid University, Abha, Saudi Arabia; ^4^Department of Pathology, Sub-division of Medical Microbiology, College of Medicine, University of Ha'il, Ha'il, Saudi Arabia; ^5^Department of Physiotherapy, College of Applied Medical Sciences, University of Ha'il, Ha'il, Saudi Arabia; ^6^Department of Child Health, College of Medicine, King Khalid University, Abha, Saudi Arabia; ^7^Department of Basic Sciences, Deanship of Preparatory Year, University of Ha'il, Ha'il, Saudi Arabia

**Keywords:** SARS-CoV-2, S1-RBD, COVID-19, ELISA, antibodies, seroprevalence

## Abstract

**Background:**

Two years into the pandemic, yet the threat of new SARS-CoV-2 variants continues to loom large. Sustained efforts are required to fully understand the infection in asymptomatic individuals and those with complications. Identification, containment, care, and preventative strategies rely on understanding the varied humoral immune responses.

**Methods:**

An in-house ELISA was developed and standardized to screen for serum IgG antibodies against the SARS-CoV-2 S1-RBD protein as an antigen. This study aims to investigate the seroprevalence of serum antibodies against S1-RBD antigen in pre-pandemic (*n* = 120) and during the early pandemic period (*n* = 120) in subjects from the Hail region, KSA and to correlate it with clinical and demographic factors.

**Results:**

Samples collected from both male (*n* = 60) and female (*n* = 60) subjects during the pandemic in the age groups of 20–40 (0.31 ± 0.029 and 0.29 ± 0.024, respectively) and 41–60 years (0.35 ± 0.026 and 0.30 ± 0.025, respectively) showed significantly higher levels of serum antibodies against S-RBD antigen than the age-matched pre-pandemic samples [male (*n* = 60) and female (*n* = 60)]. Pandemic subjects exhibited significantly (*p* < 0.01) higher inhibition (80–88%) than age-matched pre-pandemic subjects (32–39%). Antibodies against S1-RBD antigen were detected in approximately 10% of the total pre-pandemic population (males and females). However, subjects > 60 years did not show antibodies.

**Conclusion:**

Antibody levels increased in samples collected during the pandemic, even though these subjects were not clinically COVID-19 positive. A small number of pre-pandemic subjects showed serum antibodies, suggesting prior exposure to other coronaviruses in the region. With dwindling neutralizing antibody levels and reduced vaccine efficacy against newer variants, it remains crucial to develop better assays for surveillance, management, and future research.

## Introduction

The end of 2019 witnessed the emergence, rise, and rapid spread of a highly contagious novel coronavirus known as Severe Acute Respiratory Syndrome Coronavirus-2 (SARS-CoV-2), the causative pathogen of the highly contagious Corona Virus Disease 2019 or COVID-19, to almost every corner of the world (1). COVID-19 continues to be a threat, with the possible emergence of new variants with the ability to spread more rapidly and target children. Factors such as gender, age, and comorbid conditions contribute to disease severity and complications (2). The repercussions of this health crisis will be felt for many years to come.

The phylogenetically similar coronaviruses—SARS-CoV-2, Severe Acute Respiratory Syndrome Coronavirus (SARS-CoV), and the Middle East Respiratory Syndrome Coronavirus (MERS-CoV)—are beta coronaviruses, emergent from animal reservoirs, capable of rapid transmission and serious infectious outcomes in humans (3). The primary mode of COVID-19 virus transmission responsible for the pandemic is human-to-human, *via* aerosols and droplets, from infected individuals through talking, coughing, or sneezing (4). COVID-19 has a probable asymptomatic incubation period between 2 and 14 days, with newer variants displaying even lower incubation periods (5).

Those infected with the virus can broadly be classified according to their level of infection and the severity of the disease. Some infected individuals remain asymptomatic, whereas others experience mild, transient symptoms. A substantial number of infected individuals with advanced age and medical comorbidities such as diabetes, hypertension, or immunocompromised states are hospitalized due to complications (2). Depending on their immunological condition, individuals infected with COVID-19 experience mild, moderate, or severe symptoms. Common symptoms include dry cough, fever, fatigue, loss of taste or smell, and diarrhea. Severe symptoms include dyspnea and chest pains (6). Severe pathological manifestations of the disease in the infected population with comorbidities include acute respiratory distress syndrome (ARDS) and respiratory failure (7). Thus, age, pre-comorbidities, an increased viral load, low SARS-CoV-2 antibody response, or an excessive systemic inflammatory response known as a cytokine storm are contributory risk factors to adverse patient outcomes (2, 8).

SARS-CoV-2 is an enveloped virus with a linear, unsegmented positive-sense RNA genome. The nucleocapsid of the virion consists of N-phosphoprotein (NP) and RNA, surrounded by lipid bilayers (9). The (S1) spike glycoprotein peplomer mediates viral attachment, followed by membrane fusion. This glycoprotein is immunogenic and hence the target of IgM and IgG humoral circulating antibodies (Abs) (10, 11). SARS-CoV-2 infected patients produce antibodies 4–8 days post-onset (12). Recent studies suggest the role of serum antibodies, memory B cells, and cross-reactive T-cells in conferring immune protection against the virus (13). However, more region-specific studies are needed to ascertain host vulnerability, the nature of immune responses in individuals, and the extent and duration of protection.

In spite of a plethora of primary studies conducted during the pandemic about serum immunoglobulin G (IgG) generated against S1-RBD in COVID-19, details about the prevalence, durability, response, and degree of the conference of immunity from previous infections remain understudied. In a study investigating anti-S1RBD IgG in COVID-19 hospital patients during the early pandemic with a commercial ELISA, it was found that median OD values were to be higher in patients with the severe disease than those with the mild, moderate and critical disease. However, the same pattern was not observed with respect to anti-NP IgG (14). Also, the same study found that anti-S1RBD IgG levels remained stably above positive threshold values in patients with severe infections but were lower in patients with mild or moderate infections (14). In a separate cross-sectional study of unvaccinated U.S. adults, anti-S1RBD antibodies were detected in 99% of individuals who reported a positive COVID-19 test, 55% of individuals who believed they had COVID-19 but were not tested, and 11% of individuals who believed that they never had a COVID-19 infection. Also, in individuals with a positive COVID-19 result, anti-RBD levels were detectable for up to 20 months (15).

The current study aims to investigate the seroprevalence of serum antibodies against SARS-CoV-2 in the general population before the emergence of the virus and during the early phase of the COVID-19 pandemic, using an ELISA designed to screen IgG antibodies directed against viral S1-glycoprotein receptor-binding domain (S1-RBD) protein antigen. It is a crucial first step in determining the humoral immune response of asymptomatic and subclinical infections in individuals and their associated implications. Such information is vital for both researchers and policymakers in developing successful surveillance and management strategies for vaccine delivery, care of unvaccinated and vaccinated infected individuals, and effective age-related outcomes.

## Materials and Methods

### Study Subjects–Sera Collection Pre-pandemic and During COVID-19 Pandemic

A total of 240 sera samples were collected from healthy individuals before and during the early months of the COVID-19 pandemic from the Hail region, Saudi Arabia, with their prior consent. The research study was carried out per the Declaration of Helsinki (1964). Of the samples collected, 120 sera samples were from individuals who were not diagnosed with any disease. Furthermore, individuals with immune disorders, immunodeficiencies, allergies, cancer, pregnant women, and those with serious lung, heart, kidney, or liver disease were excluded from the study. An equal number of sera samples were collected from individuals during the COVID-19 pandemic with no history of COVID-19 infection and no administration of any COVID-19 vaccine. Samples were collected under the Research Ethics Committee; the University of Hail approved the study protocol H-2021-122. Subjects with any previous history of disease or associated complications, including COVID-19, were excluded from this study. Serum samples were kept in temperature-controlled environments (-20 to−80°C). Demographic data collected for the sera samples included age, gender, fasting blood glucose (FBG), basal metabolic rate (BMR), and smoking history. FBG, HbA1c, and BMR were assessed using well-known methods prescribed regularly in the clinics. Participants were asked to report symptoms such as fever, fatigue, cough, or myalgia in the 14 days prior to sample collection, as these may assist in the interpretation of antibody results.

The participants in this study were divided into groups, each comprising 20 volunteers (*n* = 20), assorted in both gender and age. The demographic data of the groups are represented in Table 1. The distribution is as follows: serum samples collected from men pre-pandemic and aged 20–40 years old (M-Pre-P, 20–40 years); serum samples collected from men during the pandemic, aged 20–40 years old (M-Pan, 20–40 years); serum samples collected from women pre-pandemic aged 20–40 years old (F-Pre-P, 20–40 years); serum samples collected from women during the pandemic aged 20–40 years old (F-Pan, 20–40 years); serum samples collected from men pre-pandemic who were 41–60 years old (M-Pre, 41–60 years); serum samples collected from men during the pandemic who were 41–60 years old (M-Pan (41–60 years); serum samples collected from women pre-pandemic who were 41–60 years old (F-Pre-P, 41–60 years); serum samples collected from women during the pandemic who were aged 41–60 years (F-Pan, 41–60 years); serum samples collected from men pre-pandemic who were more than 60 years old (M-Pre-P, >61 years); serum samples collected from men during the pandemic who were more than 60 years old (M-Pan, >61 years); serum samples collected from women pre-pandemic who were more than 60 years old (F-Pre-P > 61 years) and serum samples collected from women during the pandemic who were more than 60 years old (F-Pan, > 61 years).

**Table 1:** Group characteristics of study population.

**Groups (Age in Years)** ***n =* subjects**	**Fasting blood glucose (mg/dl)**	**HbA1c (%)**	**BMR** (**cal/sq.m/hr)**	**Number of smokers (duration; years ±SD)**	**Number of subjects with**			
					**Fever**	**Fatigue**	**Cough**	**Myalgia**
M-Pre-P (20-40) *n =* 20	85.2 ± 5.4	5.5 ± 0.3	37.6 ± 2.3	8 (9.4 ± 5.1)	1	2	—	—
M-Pan (20-40) *n =* 20	83.0 ± 7.1	5.4 ± 0.3	38.7 ± 2.1	7 (8.2 ± 4.2)	4	3	3	2
F-Pre-P (20-40) *n =* 20	84.3 ± 8.3	5.5 ± 0.3	33.6 ± 3.3	−3 (4.1 ± 4.2)	1	2	1	—
F-Pan (20-40) *n =* 20	84.6 ± 5.3	5.6 ± 0.4	35.0 ± 2.9	−3 (6.3 ± 2.1)	1	2	2	—
M-Pre-P (41-60) *n =* 20	89.1 ± 7.1	5.5 ± 0.4	35.7 ± 2.6	−9 (12.6 ± 6.3)	1	2	1	—
M-Pan (41-60) *n =* 20	89.6 ± 8.5	5.6 ± 0.4	41.8 ± 3.2	−7 (15.4 ± 6.4)	4	3	4	2
F-Pre-P (41-60) *n =* 20	88.9 ± 7.3	5.4 ± 0.3	31.2 ± 3.1	−3 (11.7 ± 3.4)	—	2	1	—
F-Pan (41-60) *n =* 20	90.1 ± 7.9	5.6 ± 0.3	33.0 ± 2.7	−4 (7.4 ± 2.6)	2	2	2	—
M-Pre-P (>60) *n =* 20	97.0 ± 11.3	5.8 ± 0.5	33.9 ± 2.4	7 (22.3 ± 5.8)	—	2	2	—
M-Pan (>60) *n =* 20	98.3 ± 9.5	5.7 ± 0.3	39.9 ± 2.8	7 (26.4 ± 4.8)	6	6	6	3
F-Pre-P (>60) *n =* 20	96.8 ± 8.8	5.7 ± 0.4	31.0 ± 2.6	4 (13.1 ± 3.9)	1	2	1	—
F-Pan (>60) *n =* 20	97.2 ± 8.8	5.8 ± 0.5	36.7 ± 3.4	4 (16.1 ± 3.9)	3	3	2	1

*M-Pre-P, M-Pan, F-Pre-P and F-Pan represents Male subjects' pre-pandemic, Male subjects during pandemic, Female subjects' pre-pandemic and female subjects during pandemic, respectively. Normal ranges for FBG are 70-99 mg/dl. Normal ranges for BMR adult men and women are 35–38 and 32–35 cal/sq.m/hr, respectively*.

### Estimation of Inflammatory Cytokines IL-6 and TNF-α

Cytokines IL-6 and TNF-α levels were analyzed in serum samples from all the cohorts using commercially available quantitative sandwich immunoassay kits (R&D System, Minneapolis, MN, USA). The sensitivities of the ELISA kits were <0.5 pg/mL. Samples were assayed in triplicate.

### Optimization of Antigen Concentration for Indirect Binding ELISA

The recombinant S1-RBD-protein antigen (MyBioSource, USA) coating concentration was optimized as described previously with slight modifications (16, 17); varying concentrations 0.1, 1, 2, 4, 8, and 10 μg/ml) of S1-RBD protein in coating buffer (0.05 M carbonate-bicarbonate buffer, pH 9.6) was coated on the ELISA plate. The plate was incubated for 2 h, and unbound antigens were removed by washing using phosphate buffer saline (PBS). Unbound spaces were blocked with 2.5% BSA and incubated for 1 h at 37°C. The ELISA plate was washed three to five times with PBS-Tween20 (PBS-T). Test samples [anti-R-C19-S1-RBD IgG (MyBioSource, California, USA)] and serum samples from three COVID-19 convalescent patients were diluted (1:100) in dilution buffer [phosphate buffer saline (PBS)] and were added to each well (100 μl/well). The plates were incubated at 37°C for 2 h, and after incubation, the ELISA plate was washed three to five times with PBS-T. The secondary antibody against anti-R-C19-S1-RBD IgG (MyBioSource, California, USA) was diluted as per the manufacturer's instruction to 1:50,000 and added to the ELISA plate (100 μl/well). For serum samples, secondary anti-human IgG diluted 1:2,000 in dilution buffer was added to each well (100 μl/well). Secondary antibodies were conjugated to horseradish peroxidase (GE Healthcare). After incubation at 37°C for 2 h, the plate was washed three to five times with PBS-T. 3,3',5,5'-Tetramethylbenzidine (TMB). Stabilized substrate (Promega) was added to each well (100 μl) for 20 min. One molar of H_2_SO_4_ (100 μl) was used to stop the enzyme reaction after 20 min incubation at room temperature. The results were expressed as optical density (OD) (OD = mean of triplicate wells minus mean of the blank wells). The OD of the reaction product was read at 450 nm on an ELISA plate reader.

### Optimization of Serum Dilution for Indirect Binding ELISA

Serum dilution from three COVID-19 convalescent patients was optimized for the ELISA assay. The ELISA plate was coated with 2 μg/ml of S1-RBD antigen in coating buffer and incubated for 2 h. Unbound antigens were removed by washing with PBS. Unbound spaces in the ELISA plate were blocked with 2.5% BSA and incubated for 1 h at 37°C. The plate was washed three to five times with PBS-T. Serum samples (*n* = 3) diluted serially (1:50, 1:100, 1:200, 1:400, 1:800, and 1:1,600) in PBS were added to each well (100 μl/well). The plates were incubated at 37°C for 2 h and, after incubation, washed three to five times with PBS-T. Secondary anti-human IgG conjugated to horseradish peroxidase was diluted at 1:2,000 in a dilution buffer and added to each well (100 μl/well). The remaining steps were the same as those given above.

### Determination of the Threshold Value by Indirect ELISA

The OD450 nm value of 20 sera samples of normal individuals from our laboratory, obtained before the COVID-19 outbreak, was detected with the optimum concentration of protein and antibody by indirect ELISA. The results were statistically analyzed to determine the cut-off value. The mean (X) and standard deviation (SD) of the 20 samples were calculated. The cut-off value was X + 3 SD, which was positive when the IgG OD450 nm value of the samples to be tested was ≥X + 3 SD and negative when the IgG OD450 nm value was < X + 3 SD.

### ELISA Plate Description

To test the sera samples, the following design of a 96-well ELISA plate was used: twenty serum samples from each group were tested in triplicate on each plate along with antibody specific (anti-R-C19-S1-RBD IgG) for S1-RBD antigen, which served as a positive control (three wells). Six wells included two different pre-pandemic serum samples that showed <0.1 OD in indirect binding ELISA and were considered negative controls. Blanks were also included in three wells. The results were expressed as OD (OD = mean of triplicate wells minus the mean of the blank wells).

### Isolation of Serum IgG

Affinity chromatography was applied to isolate IgG from serum samples using a Protein A-Agarose column (Sigma-Aldrich, USA). The Protein A-Agarose column was washed 2–3 times using PBS buffer (pH 7.4) prior to the addition of the sample. A volume of 0.5 ml of serum sample was diluted with an equal volume of PBS (pH 7.4) and run through the column. Samples were re-eluted 2–3 times for efficient binding of IgG. Unbound IgG was removed by extensive washing with the same washing buffer. Serum IgG, which was bound to the column, was eluted with elution buffer (acetic acid (0.58%) in sodium chloride (0.85%) and neutralized with 1 M Tris-HCl (pH 8.5). About 2–3 ml of fractions were collected in serum tubes, and each tube was read at 251 and 278 nm. The concentration of IgG was estimated as 1 mg/ml at 1.4 OD.

### Specificity and Reproducibility of Indirect ELISA

The specificity of the indirect binding ELISA was assessed by evaluating the presence of SARS-CoV-2 specific IgG against S1-RBD in the sera samples of COVID-19 convalescent patients (*n* = 3), hepatitis C virus (HCV) (*n* = 3), tuberculosis (TB) (*n* = 3), and rheumatoid arthritis (RA) (*n* = 3) patients. The ELISA plates were coated with 2 g/ml of S1RBD antigen, and the specificity of the method was evaluated with the established indirect ELISA method (15, 16). Anti-R-C19-S1-RBD IgG served as a positive control, and IgG from pre-pandemic subjects who showed <0.2 OD served as a negative control.

Plate-to-plate variation was monitored by comparing the control panel results between the different wells of the same plate; the same sera samples were run on different plates on the same day as well as on different days.

### Indirect Binding ELISA

The binding activity of serum antibodies to S1-RBD antigen was detected by indirect binding ELISA as described above with slight modifications (16–19).

### Inhibition ELISA

The specificities of S1-RBD antigen and serum IgG were estimated by competition ELISA (18–20). Increasing concentrations of S1-RBD antigen (0–10 μg/ml) were allowed to interact with a constant amount of serum autoantibodies from individuals of different groups for 2 h at room temperature and overnight at 4°C. After incubation, the immune complex formed was incubated in the microplate wells (instead of the serum taken in indirect binding ELISA), and the bound antibody levels were detected as in indirect binding ELISA. The percent inhibition was calculated using the formula:


$$\text{Percent inhibition=}[\text{1-}({\text{A}}_{\text{inhibited}}{\text{/A}}_{\text{uninhibited}})]\times \text{100},$$

where A_inhibited_ is the absorbance at 10 μg/mL of inhibitor concentration and A_uninhibited_ is the absorbance at zero inhibitor concentration.

### Statistical Analysis

Statistical analyses were carried out using OriginPro v6.1. One-way or two-way analysis of variance (ANOVA) was applied to test for statistical significance. Only *p*-values of 0.05 or lower were considered statistically significant [*p* > 0.05 (ns, not significant), *p* ≤ 0.05 (^*^), p ≤ 0.01 (^**^), *p* ≤ 0.001 (^***^)].

## Results

### Optimization of Antigen for ELISA

**Figure 1:**
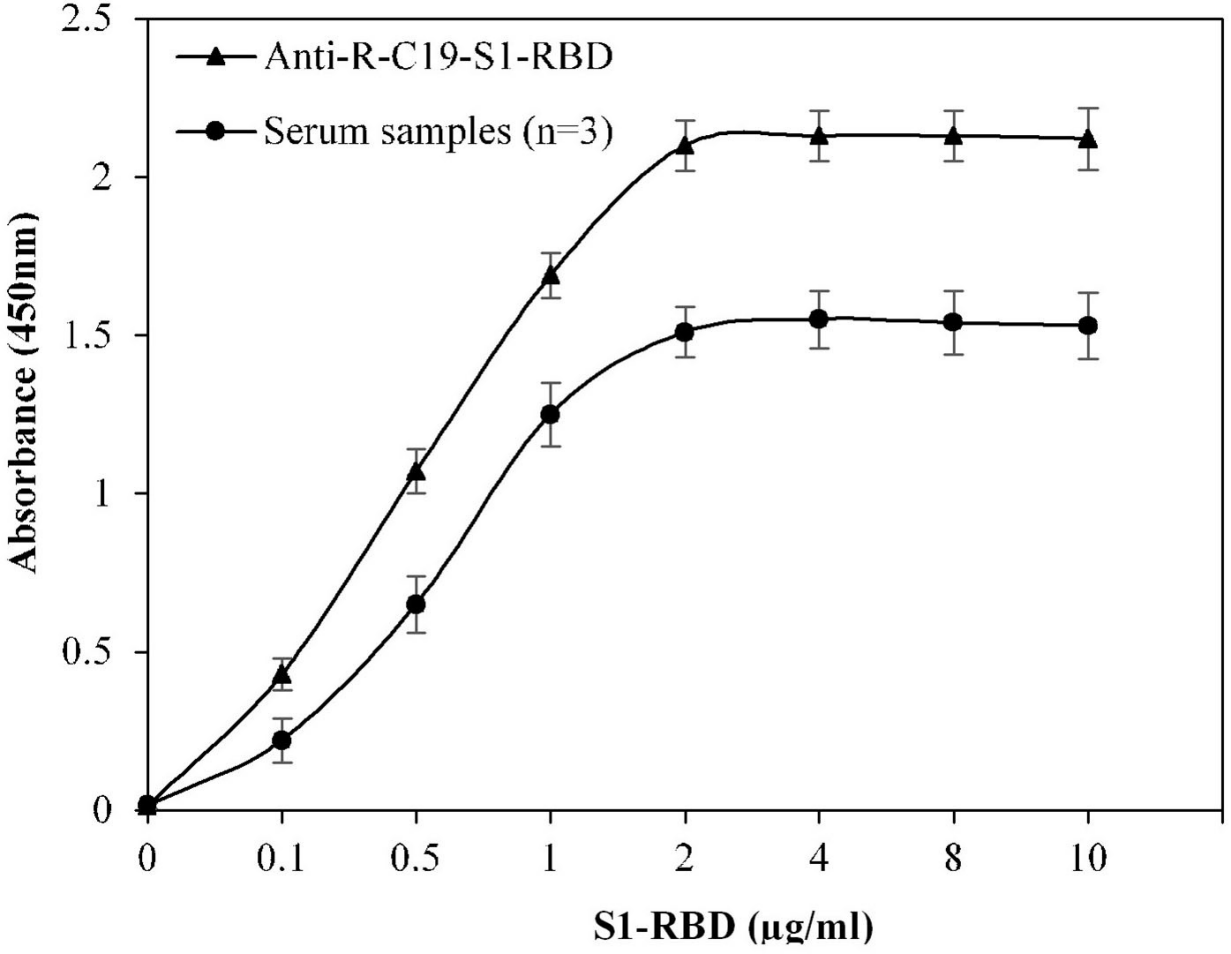
Optimization of antigen (S1-RBD) concentration for ELISA. Serum samples from COVID-19 convalescent individuals (*n* = 3) were used in the assay. Anti-R-C19-S1-RBD IgG was used a positive control. Each sample was run in triplicate under similar conditions.

Multiple steps were included in the ELISA method. To develop an efficient ELISA assay, it is essential to standardize all steps. The concentration of S1-RBD protein antigen used to coat the microplate was optimized, which effectively covered the bottom of the microplate wells. Figure 1 shows that at a concentration of 2 μg/ml, recombinant S1-RBD protein antigen exhibited maximum absorbance, which was recorded for both anti-R-C19-S1-RBD IgG and serum samples. Hence, 2 μg/ml of recombinant S1-RBD protein antigen was used for all further ELISA assays.

### Optimization of Serum Dilution for ELISA

**Figure 2:**
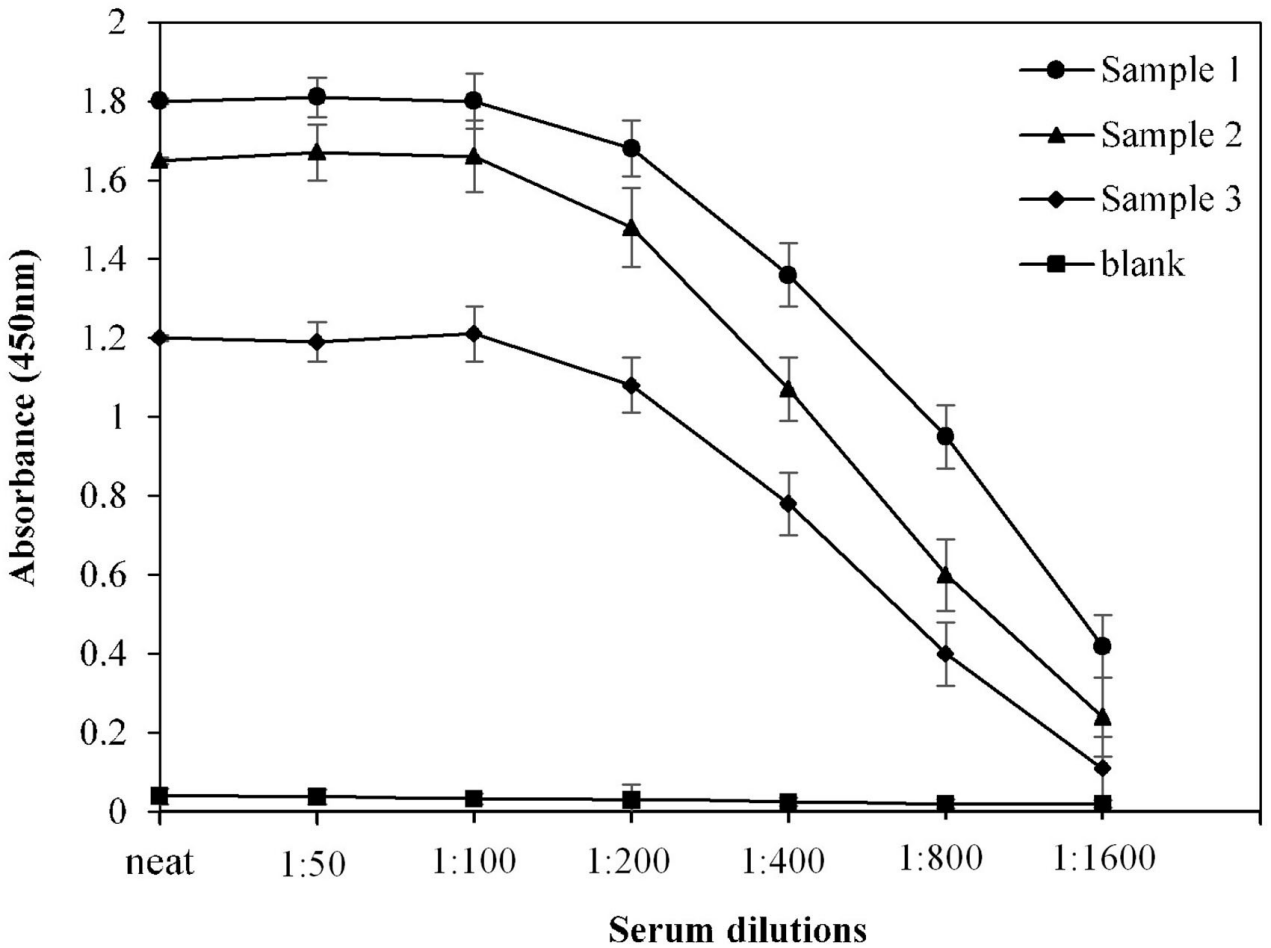
Optimization of serum dilution for ELISA. Serum samples from COVID-19 convalescent individuals (*n* = 3) were used in the assay. Each sample was run in triplicate under similar conditions.

For optimization of serum dilution used in indirect binding ELISA, serum samples from three COVID-19 convalescent patients were diluted with varying ratios. Maximum absorbance against commercially available S1-RBD protein antigen was observed at neat as well as at 1:50 and 1:100 dilutions (Figure 2). Therefore, for further ELISA assays, a serum dilution of 1:100 was used.

### Determination of the Threshold Value by Indirect ELISA

**Figure 3:**
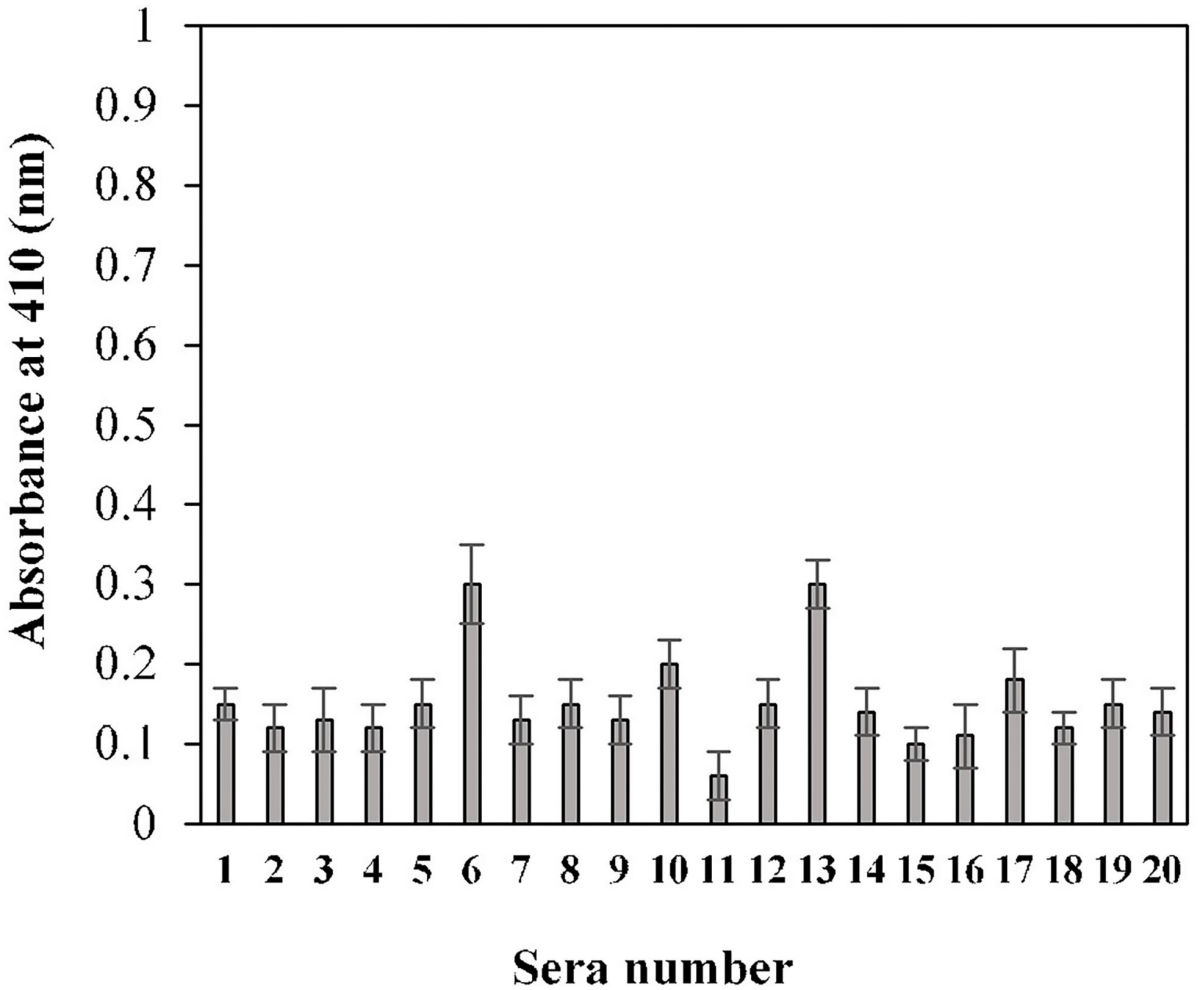
Estimation of cut-off value for indirect binding ELISA. Pre-pandemic serum samples (*n* = 20) were used in the ELISA (1:100 dilution) assay against the S1-RBD antigen (2 μg/ml). Each sample was run in triplicate.

The calculated cut-off value, using the given method for the 20 randomly selected pre-pandemic normal sera samples, was found to be 0.2 (OD) (Figure 3). An in-house threshold or cut-off ratio value, which best distinguished elevated anti-S1-RBD antibody levels from healthy control individuals, was established to be 0.2.

### Specificity and Reproducibility of Indirect ELISA

**Figure 4:**
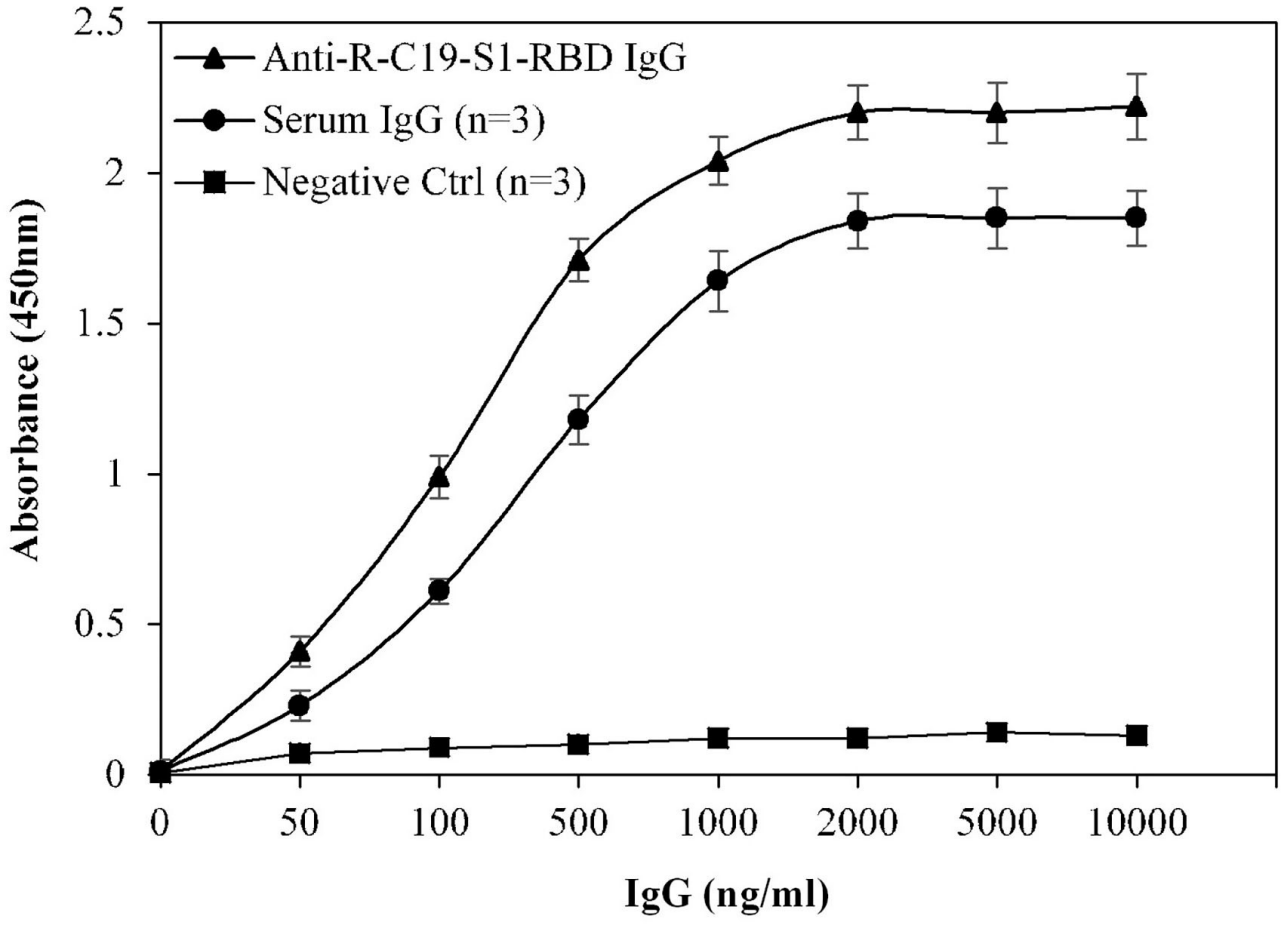
Titration curves for the optimization of serum IgG (COVID-19 patients; *n* = 3). Commercially available anti-R-C19-S1-RBD IgG was used as a positive control. Three nonreactive serum samples from pre-pandemic subjects served as a negative control. ELISA plates were coated with an antigen (S1-RBD) concentration of 2 μg/ml. Each sample was run in triplicate under similar conditions.

Specificity of the assay was investigated using a titration assay of R-C19-S1-RBD IgG, purified IgGs from COVID-19 convalescent patients (*n* = 3), and pre-pandemic serum (*n* = 3) (Figure 4). At a concentration of 2,000 ng/ml, COVID-19 convalescent patients' IgG exhibited higher specificity (1.84 ± 0.09; *p* <0.0001) than pre-pandemic subjects' IgG (0.12 ± 0.10). R-C19-S1-RBD IgG served as a positive control (2.20 ± 0.09).

**Figure 5:**
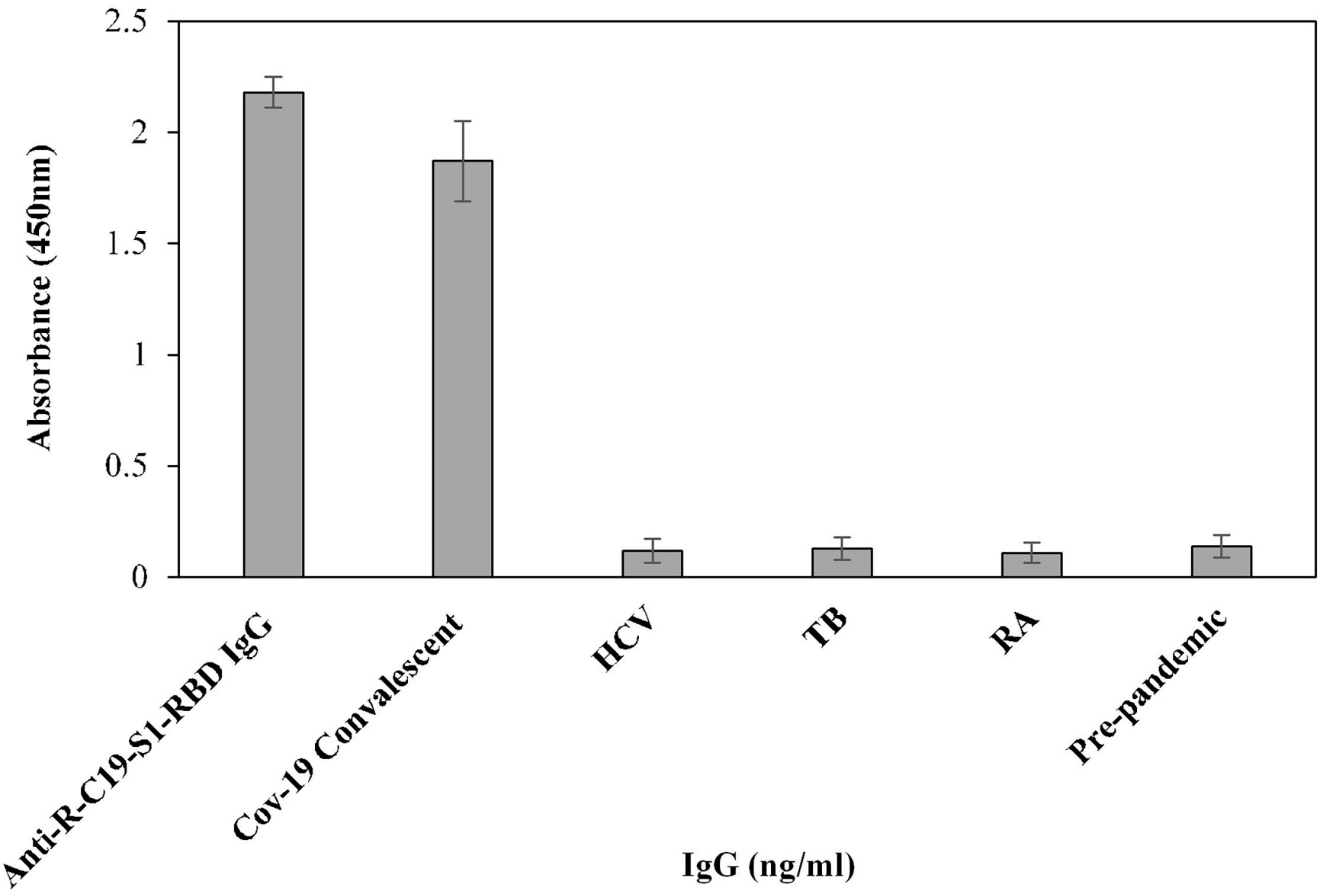
The specificity of the indirect binding ELISA was established by screening three serum samples each from COVID-19 convalescent, HCV, TB and RA patients. Anti-S1-RBD-IgG and pre-pandemic subjects (*n* = 3) were used as positive and negative controls, respectively.

Furthermore, the cross-reactivity of serum IgG was investigated using isolated serum IgGs (*n* = 3) from patients with HCV, TB, and RA, showing negligible binding (0.12 ± 0.054, 0.10 ± 0.05 and 0.11 ± 0.045, respectively) (Figure 5). However, serum IgG from COVID-19 convalescent patients (OD: 1.87 ± 0.18) exhibited significantly higher (*p* <0.0001) binding compared to HCV, TB, RA, and blank (Figure 5). Anti-R-C19-S1-RBD IgG (2.18 ± 0.07) and IgG from pre-pandemic subjects (0.13 ± 0.05) served as positive and negative controls, respectively.

### Inflammatory Cytokine Levels

**Figure 6:**
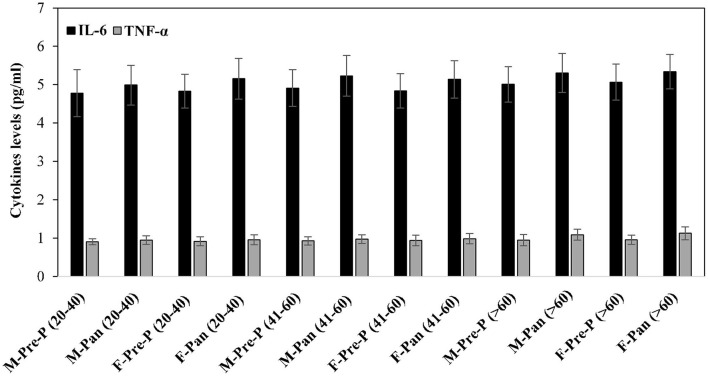
Serum inflammatory cytokines TNF-α and IL-6 levels (pg/ml) were estimated from all the studied groups. All samples were in triplicates, and values are given as mean ± SD.

Inflammatory cytokines such as IL-6 and TNF-α were estimated in serum samples of all the subjects from different groups (Figure 6). Post-pandemic subjects exhibited slightly elevated levels of IL-6. However, these differences were non-significant. No remarkable changes were observed in TNF levels in all groups.

### Clinical and Epidemiological Characterizations

Clinical and epidemiological data for 240 sera samples from different groups assorted by age and gender are presented in Table 1. Volunteers included equal numbers of men and women in each group. FBG levels were within the normal range (70–98 mg/dl) for all groups. However, increased FBG levels were observed in older age groups (>60 years) as compared to other age groups (<60 years). HbA1c was found to be within the normal range; however, slightly elevated levels (non-significant) were observed in subjects aged more than 60 years. BMR for pandemic groups showed slightly increased values compared to pre-pandemic groups. In women, the BMR was lower than in men of corresponding age groups. Significantly high BMR was found in groups [M-Pan (20–40, *p* < *0.05*), M-Pan (41–60, *p* < *0.01*), M-Pan (>60, *p* < *0.01*), and F-Pan (>60, *p* < *0.05*)] in which subject(s) showed symptoms of fever, cough, and myalgia altogether (Table 1). Additionally, this trend was observed only in groups with a higher number of smokers and with an increased smoking duration.

### Indirect Binding ELISA

The binding efficiency of serum antibodies and S1-RBD protein antigen was evaluated for all age and gender assorted groups. Serum samples were tested at a dilution of 1:100 in an indirect binding ELISA against the S1-RBD antigen (2 μg/ml).

**Figure 7:**
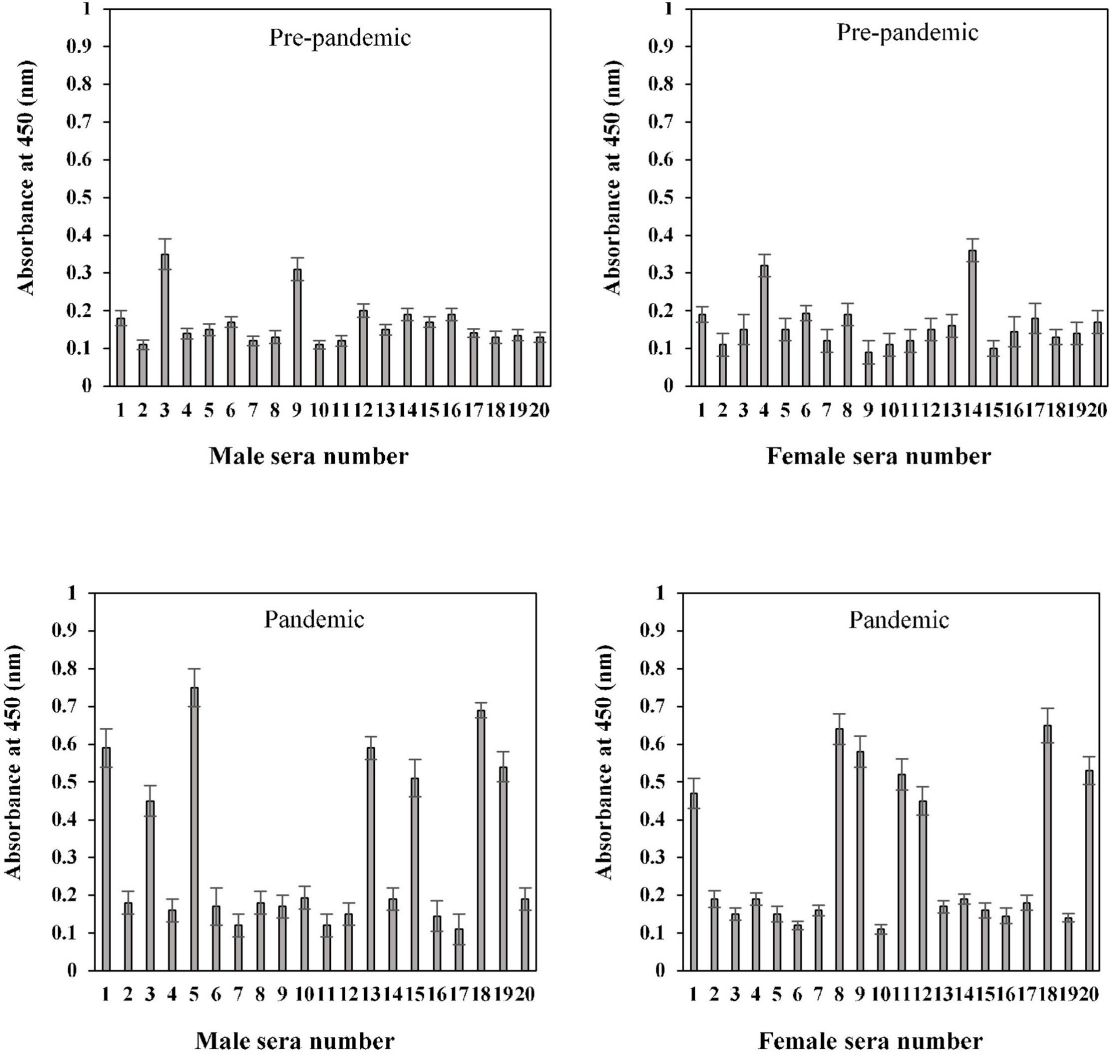
Indirect binding ELISA of pre-pandemic samples and samples collected during the pandemic from men and women aged 20–40 years. ELISA plates were coated with an antigen concentration of 2 μg/ml. Each sample was run in triplicate under similar conditions.

The binding specificities of serum antibodies against the S1-RBD antigen in samples collected before and during the COVID-19 pandemic were found to vary among the 20–40-year-old age group subjects. Samples collected from both male and female subjects pre-COVID showed low binding toward antigen, i.e., 0.17 ± 0.016 and 0.16 ± 0.018, respectively. However, significantly (*p* < 0.05) higher binding was observed in sera samples collected during the pandemic from subjects of both genders (male and female) corresponding to the same age groups, 0.31 ± 0.029 and 0.29 ± 0.024, respectively (Figure 7). Only two pre-pandemic serum samples, each from men and women aged 20–40 years, were found to be positive (average values; 0.33 ± 0.024 and 0.34 ± 0.027, respectively). However, for the same age and gender-matched samples collected during the pandemic, seven samples were found to be positive for men and women (average values; 0.59 ± 0.039 and 0.55 ± 0.035, respectively) each (Figure 7).

**Figure 8:**
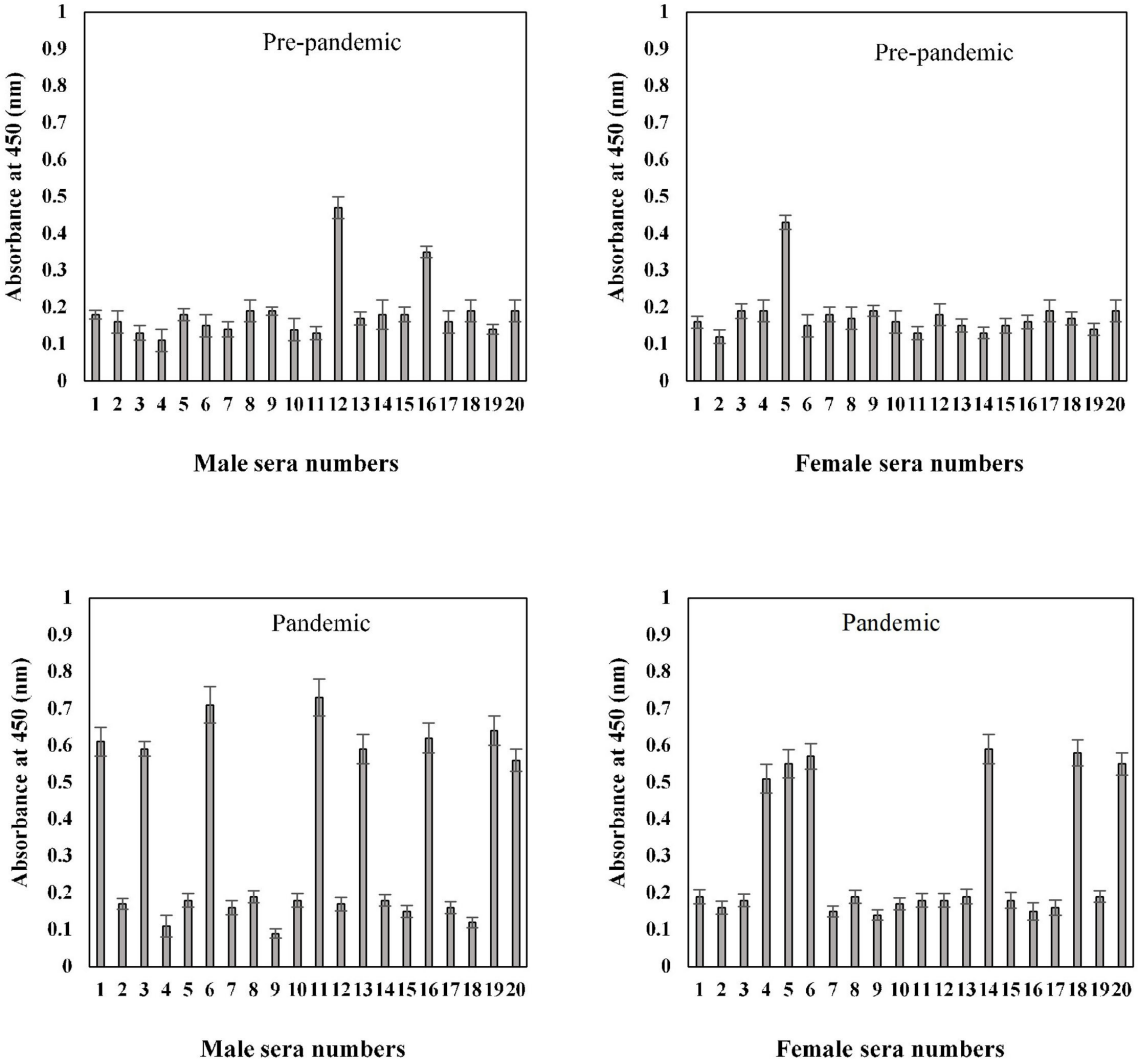
Indirect binding ELISA from samples collected pre-pandemic and during the pandemic from men and women aged 41–60 years. ELISA plates were coated with an antigen concentration of 2 μg/ml. Each sample was run in triplicate under similar conditions.

Serum antibody binding patterns against the S1-RBD antigen were evaluated for both male and female subjects aged 41–60 years (Figure 8). Low levels of binding were observed in both male (0.19 ± 0.018) and female (0.18 ± 0.017) subjects from serum samples collected before the start of the pandemic. Samples collected during the pandemic from both male and female individuals exhibited significantly (*p* < 0.05) higher binding (0.35 ± 0.026 and 0.30 ± 0.025, respectively) against the antigen as compared to the age-matched pre-pandemic subjects. From samples collected during the pandemic in the age group of 41–60 years, eight samples from men (0.63 ± 0.041) and six samples from women (0.59 ± 0.043) showed positive binding with high reactivity (Figure 8). Comparatively, a much smaller number of pre-pandemic samples showed positive binding and reactivity; two samples were from men (0.41 ± 0.033) and one sample (0.43 ± 0.032) from women.

**Figure 9:**
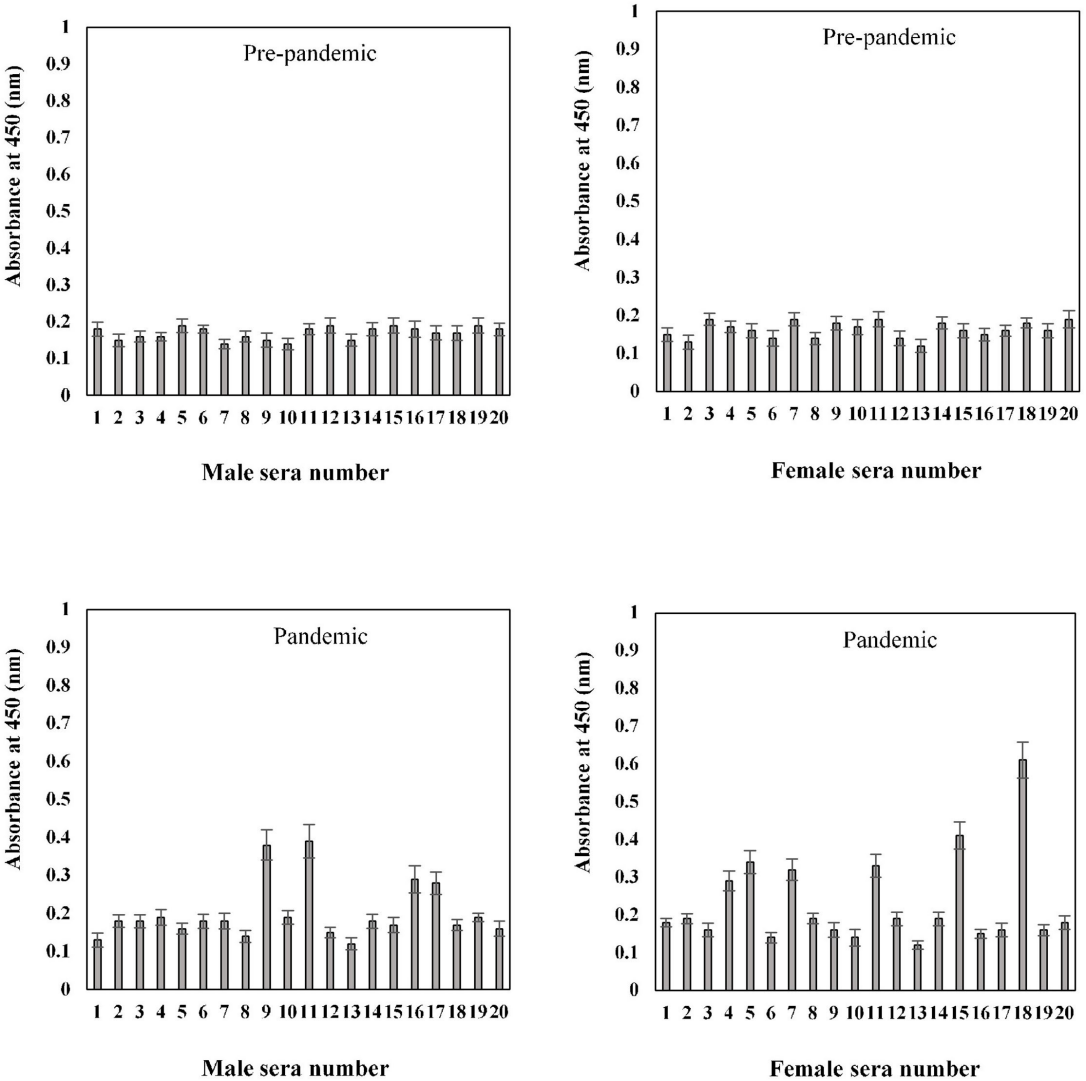
Indirect binding ELISA from samples collected pre-pandemic and during the pandemic from men and women aged > 60 years. ELISA plates were coated with an antigen concentration of 2 μg/ml. Each sample was run in triplicate under similar conditions.

In the sample group > 60 years of age, no binding activity was detected among both male (0.17 ± 0.017) and female (0.16 ± 0.017) subjects in samples collected prior to the pandemic (Figure 9). Samples for both men (0.20 ± 0.022) and women (0.23 ± 0.021) collected during the pandemic showed less reactivity toward the antigen. However, in the same age group, seropositivity was detected in four men (0.285 ± 0.027) and six women (0.38 ± 0.039) samples collected during the pandemic. Moreover, the positive samples from > 60 years olds showed a low level of reactivity when compared to positive samples from other groups (20–40 and 41–60 years) collected during the pandemic.

### Correlation Analysis

Correlation analysis was performed for all pre-pandemic samples as well as samples collected during the pandemic for antibodies against S1-RBD in different age groups and various parameters (FBG, BMR, smoking, fever, fatigue, cough, and myalgia) (see Table 2). This analysis showed that the data for fever, fatigue, cough, and myalgia significantly correlated with antibodies against S1-RBD in samples collected during the pandemic in higher age groups (41–60 and >60) for both male and female subjects. However, for the age group 41–60 years, more parameters showed a correlation for male subjects [M-Pan (41–60)] as compared to female subjects [F-Pan (41–60)]. Parameters such as fever, fatigue, and cough consistently correlated with samples collected during the pandemic for all groups. FBG levels did not exhibit a correlation with any of the groups. Correlation analysis showed that myalgia could be strongly correlated with circulating IgG against COVID-19 infection, even though subjects had not been diagnosed with the disease.

**Table 2:** Correlation between anti-S1-RBD antibody levels and study parameters in various age groups.

		**M-Pre-P** **(20-40)**	**M-Pan** **(20-40)**	**F-Pre-P** **(20-40)**	**F-Pan** **(20-40)**	**M-Pre-P** **(41-60)**	**M-Pan** **(41-60)**	**F-Pre-P** **(41-60)**	**F-Pan** **(41-60)**	**M-Pre-P** **(>60)**	**M-Pan** **(>60)**	**F-Pre-P** **(>60)**	**F-Pan** **(>60)**
		**r**	**r**	**r**	**r**	**R**	**r**	**r**	**r**	**r**	**r**	**r**	**r**
Antibodies Against	FBG	0.05	0.32	0.37	0.1	0.35	0.05	0.07	0.04	0.15	0.39	0.23	0.1
S1-RBD	BMR	0.08	0.61*	0.44	0.25	0.13	0.77**	0.28	0.38	0.2	0.71**	0.16	0.64*
	Smoking	0.13	0.34	0.05	0.43	0.23	0.91***	0.67*	0.21	0.4	0.63*	0.3	0.76**
	Fever	0.03	0.6*	0.19	0.42	0.04	0.68*	NA	0.6*	NA	0.87***	0.03	0.81***
	Fatigue	0.09	0.67*	0.3	0.61*	0.02	0.60*	0.11	0.44*	0.1	0.71**	0.12	0.74**
	Cough	NA	0.67*	0.26	0.61*	0.04	0.69*	0.17	0.6*	0.3	0.68*	0.19	0.68*
	Myalgia	NA	0.61*	NA	NA	NA	0.63*	NA	NA	NA	0.86***	NA	0.74**
	IL-6	0.06	0.08	0.07	0.08	0.06	0.09	0.05	0.09	0.07	0.09	0.06	0.08
	TNF-α	0.03	0.04	0.03	0.03	0.04	0.03	0.04	0.04	0.03	0.05	0.04	0.06

Correlation analyses were performed using SPSS18. Pearson correlation tests were adopted to analyze the correlations between parameters. Significance is defined as *P < 0.05, **P < 0.001, ***P < 0.0001. NA represents not applicable.

### Inhibition ELISA of Serum Antibodies Against S1-RBD

The binding specificity of the circulating antibodies to the S1-RBD antigen was further ascertained by inhibition ELISA using S1-RBD as an inhibitor, as given in Table 3. As shown in indirect binding ELISA results, inhibition ELISA of subjects from samples collected during the pandemic showed a significantly (*p* <0.001) higher maximum percent inhibition than the age-matched subjects from the pre-pandemic group. The highest mean percent inhibition was detected in subjects from groups M-Pan (41–60), followed by M-Pan (20-40), F-Pan (20-40), M-Pan (41-60), F-Pan (>60), and M-Pan (>60) (see Table 3). In contrast, very low mean percent inhibitions (39 ± 3.4 – 32 ± 3.0) were observed in most of the groups of the pre-pandemic subjects. M-Pre (>60) and F-Pre (>60) did not have any positive samples. However, two samples were randomly selected from these groups and tested for inhibition ELISA (see Table 3).

**Table 3:** Pre- and intra-pandemic comparison of serum IgG inhibition in various age group.

**Subject groups** **(years)**	**Mean maximum percent inhibition**	
	**Pre-pandemic**	**Pandemic**
Male (20–40)	37 ± 3.1 (2)*	83 ± 4.8 (7)*
Female (20–40)	39 ± 3.4 (2)*	82 ± 4.3 (7)*
Male (41–60)	34 ± 3.8 (2)*	88 ± 5.5 (8)*
Female (41–60)	32 ± 3.0 (2)*	80 ± 5.1 (7)*
Male (≥61)	4.2 ± 1.6 (2)^♯^	43 ± 4.9 (4)*
Female (≥61)	3.9 ± 1.4 (2)^♯^	48 ± 4.6 (6)*

* *Values in parenthesis showed positive sera samples against S1-RBD antigen.*

♯ *Randomly selected non-positive samples against S1-RBD antigen. The ELISA plates were coated with antigen (2 μg/ml). Antigen was used as an inhibitor*.

This data showed that pre-pandemic subjects older than 60 years did not have antibodies when compared to subjects aged 40 years or less. This may contribute to the higher death rate in older age groups (>60 years) in COVID-19 patients. Even circulating antibodies in positive subjects belonging to older age groups (>60 years) collected during the pandemic showed less specificity for the S1-RBD antigen than in subjects aged <40 years.

## Discussion

This study highlights the potential contribution of serology in understanding the difference in the immune status of individuals before the onset of and during the early phase of the COVID-19 pandemic. We report an in-house ELISA assay, developed and optimized to detect antibodies against the S1 domain of SARS-CoV-2 spike protein characteristics of SARS-CoV2 anti-S1-RBD IgG antibodies in sera samples of individuals collected pre-pandemic and during the COVID-19 pandemic.

The preferred method for diagnosing SARS-CoV-2 infection has been through viral nucleic acid or RT-qPCR tests, which require pharynx swab samples. This method, although highly sensitive, is subject to sampling techniques. The ELISA assay is an alternate assay that is both highly specific, sensitive, and cost-effective. Not only is this method suitable for large-scale sample testing and diagnosis but it also provides valuable information about the humoral state of the subject (21, 22). This study focuses on the development of an easy-to-use and high-throughput serological ELISA method with a low threshold value, which is specific, sensitive, and reproducible. This detection method can potentially identify asymptomatic, subclinical, or prior infections. Our optimized protocol can be implemented to accommodate large-scale automated testing of COVID-19 antibodies against the S1-RBD protein antigen.

The study aimed to investigate differences in anti-S1-RBD antibody profiles in sera samples collected from equal numbers of gender and age-matched individuals pre-pandemic and during the pandemic in 2020 to discern differences in trends. Along with demographic data, it is essential to study various clinical characteristics such as fever, cough, myalgia, fatigue, FBG, and BMR levels that may affect disease prognosis (23–26). High FBG and HbA1c levels were detected in older age groups (>60 years), which is a normal pattern in the elderly (17). BMR was also found to decrease linearly with age. Women exhibited lower BMR than men of corresponding age groups. However, participants reporting symptoms of moderate fever, fatigue, cough, and myalgia had a significantly (*p* < *0.05*) higher BMR, with the trend most apparent in men with a history of smoking. They increased smoking duration (23, 24).

The binding specificity profiles of serum antibodies against the S1-RBD antigen were ascertained by indirect binding ELISA for all age and gender assorted groups, collected pre-pandemic and during the pandemic. A smaller number of subjects (10%) with low levels of circulating antibodies against S1-RBD were identified in pre-pandemic groups (>60 years). The prior exposure of these subjects to other coronaviruses like MERS-CoV cannot be ruled out. However, a higher number of non-positive pre-pandemic subjects might not have been exposed to any coronaviruses and thus did not display antibodies, which appear to be rare (27). No positive sera samples were detected in pre-pandemic samples of both men and women aged > 60 years. Age is a significant risk factor for COVID-19 infection that has been explored in many studies (28–30). Our study is in agreement with the findings of another age-structured study which showed that in individuals aged younger than 20 years of age, susceptibility to COVID infection was found to be approximately half that of adults aged over 20 years (31). Also, the incidence of clinical symptoms exhibited in infected individuals increased with increasing age. The implications of age-related susceptibility to infection and immune outcomes are essential factors in consideration of the burden of disease. These can further be used for age-structured correlation studies for a population and projections of subclinical and clinal infections.

A recent report suggested the presence of pre-existing cross-reactive antibodies to the SARSCoV2 spike in young people, including children, mainly against the S2 domain (31, 32). However, the complete etiology of the presence of these antibodies is unknown, and hence, further investigations would be necessary for protection against future SARS-related infections.

The binding specificities were significantly higher for sera collected during the pandemic from age and gender-matched subjects, indicating higher chances of exposure to the S1-RBD antigen of SARS-CoV2. A total of 38 sera samples were strongly positive, with more male sera samples found to be positive in the age groups of 20–40 and 41–60 years, perhaps due to the higher probability of exposure, the role of sex hormones in immune activation and increased age (33). These findings can also be explained by previous studies highlighting inadequate compliance with recommended hygiene measures and contact restrictions in younger age groups and men (34).

Conversely, fewer female samples showed strong positivity in the 20–40 and 41–60-year age groups compared to male samples. This finding is in agreement with previous studies, which indicate sexual dimorphism in immune response and lower neutralizing antibody titers are significantly associated with the female sex (33, 35). However, a slightly higher number of female samples were found to be positive in the age group of >60 years, indicating a more robust immune response than men. However, the reason behind this difference is not apparent and warrants further investigation. This shows that gender is an essential factor in subjects of older age groups for the presence of antibodies against the S1-RBD antigen.

Antibodies with neutralizing activity are considered necessary for protection against SARS-CoV-2 infection. Many studies have demonstrated a close correlation between anti-SARS-CoV-2 spike IgG antibody levels and neutralizing activity (27, 36), which was also shown in this study, suggesting a critical role of anti-spike antibodies in virus neutralization. The duration of persistence of these antibodies is key to devising strategies to combat newly emergent highly transmissible variants.

Strong correlations were observed for fever, fatigue, cough, and myalgia with antibodies against S1-RBD in samples collected during the pandemic (30). However, FBG, HbA1c, and BMR either did not correlate or inconsistently correlated with a few groups. Inflammatory cytokines IL-6 and TNF-α also did not show any correlation with serum IgGs against the S1-RBD antigen. Hence, these factors cannot be ignored when assessing the disease diagnosis and the level of infection. To the best of our knowledge, this is the first seroprevalence study of IgG specific for the COVID-19 virus antigen “S1-RBD” from the Hail region in KSA, providing valuable information about IgG levels in different age groups as well as genders. Interestingly, approximately <10% of subjects exhibited the presence of these IgGs in serum samples obtained prior to the COVID-19 pandemic. The reason for this remains uninvestigated, although prior exposure of the population to another SARS virus may be a possibility. The limitations of our study include the limited numbers of samples and clinical data collected for this cross-sectional study conducted during the early phase of the pandemic due to the strict health and safety policies and restrictions. Future longitudinal studies with a larger sample size would be valuable for a comprehensive comparison of data for such samples. They may provide a better understanding of social determinants and the overall humoral immune response.

RBD-specific antibodies detected in the plasma of infected patients showed potent antiviral activity in all infected individuals, suggesting a broader role of neutralizing antibodies in COVID-19 infection, which may contribute to overall vaccine design and efficacy (37). Inhibition ELISA performed for all groups exhibited low antigen-antibody binding specificities in older age group subjects than younger individuals. Seroprevalence and surveillance studies can help identify asymptomatic or subclinical infections in a population. Such studies can offer insight into the subtle differences/variations in the underlying immunological mechanisms. Knowledge of the contribution of any pre-existing immunity to SARS-CoV-2 and the role of the humoral immune response in asymptomatic and subclinical infections is vital in devising strategies for surveillance and containment. Even with the increased availability of a range of vaccines against SARS-CoV-2, the possibility of reinfection still looms large with the threat of newer and more transmissible variants and dual viral infections, partially vaccinated or unvaccinated populations, and waning immunity levels. Understanding the extent and duration of protective immunity in individuals of a population is essential for the protection of vulnerable groups and facilitating the return of society to a state of normalcy.

## Conclusion

The current study encompasses a seroepidemiological study of anti-S1-RBD antibodies in a population in the Hail region, KSA, before the start of and during the early phase of the COVID-19 pandemic. Pre-pandemic subjects >60 years exhibited about 10 percent circulating antibodies against S1-RBD antigen, which is indicative of earlier exposure to other coronaviruses. In early pandemic subjects, the percentage of anti-S1-RBD antibodies significantly increased to 35 percent. These serum antibodies showed a strong correlation with symptoms of fever, fatigue, cough, and myalgia. Higher antibody titers are significantly associated with the male sex. However, these antibodies decreased in the elderly. This ELISA assay is an important and valuable tool for screening large numbers of samples from different age groups and assessing immune status. Age-specific antibody profiles indicate the need for targeted monitoring strategies for prevention, disease management, and vaccine effectiveness. The rise of newer variants, waning antibody levels, and reduced vaccine efficacy raise concerns about the durability of responses in clinical protection.

## Data Availability Statement

The data that support the findings of this study are available on request from the corresponding author. The data are not publicly available due to privacy or ethical restrictions.

## Ethics Statement

The studies involving human participants were reviewed and approved by Research Ethics Committee (REC) at the University of Hail. The patients/participants provided their written informed consent to participate in this study.

## Author Contributions

SS, MWAK, AM, NA, and MR: formal analysis. SS and MWAK: investigation, conceptualization, data curation, and writing the original draft preparation. SS, MWAK, and MS: resources. AS, AM, MK, NA, and MR: writing-review and editing. SS: supervision and funding acquisition. SS, MWAK, AM, and MS: methodology. All authors read and approved the final manuscript.

## Funding

This research has been funded by the Scientific Research Deanship at the University of Ha'il – Saudi Arabia through project number RG-20 077.

## Conflict of Interest

The authors declare that the research was conducted in the absence of any commercial or financial relationships that could be construed as a potential conflict of interest.

## Publisher's Note

All claims expressed in this article are solely those of the authors and do not necessarily represent those of their affiliated organizations, or those of the publisher, the editors and the reviewers. Any product that may be evaluated in this article, or claim that may be made by its manufacturer, is not guaranteed or endorsed by the publisher.
